# Oxidative Modification of Biomolecules in the Nonstimulated and Stimulated Saliva of Patients with Morbid Obesity Treated with Bariatric Surgery

**DOI:** 10.1155/2017/4923769

**Published:** 2017-12-31

**Authors:** Katarzyna Fejfer, Piotr Buczko, Marek Niczyporuk, Jerzy R. Ładny, Hady R. Hady, Małgorzata Knaś, Danuta Waszkiel, Anna Klimiuk, Anna Zalewska, Mateusz Maciejczyk

**Affiliations:** ^1^Department of Conservative Dentistry, Medical University of Bialystok, M. Sklodowskiej-Curie 24A Str., 15-274 Bialystok, Poland; ^2^Department of Orthodontics, Medical University of Bialystok, M. Sklodowskiej-Curie 24A Str., 15-274 Bialystok, Poland; ^3^Laboratory of Esthetic Medicine, Medical University of Bialystok, Akademicka 3 Str., 15-267 Bialystok, Poland; ^4^Department of General and Endocrinological Surgery, Medical University of Bialystok, M. Sklodowskiej-Curie 24A Str., 15-274 Bialystok, Poland; ^5^Department of Cosmetology, Lomza State University of Applied Sciences, Akademicka 1 Str., 18-400 Lomza, Poland; ^6^Department of Physiology, Medical University of Bialystok, Mickiewicza 2C Str., 15-222 Bialystok, Poland

## Abstract

Morbid obesity leads to progressive failure of many human organs and systems; however, the role of oxidative damage to salivary composition is still unknown in the obese patients. In this study, we assessed the effect of bariatric surgery on oxidative damage in nonstimulated (NS) and stimulated (S) whole saliva. The study included 47 subjects with morbid obesity as well as 47 age- and gender-matched healthy volunteers. Oxidative modifications to lipids (4-hydroxynonenal (4-HNE) and 8-isoprostanes (8-isoP)), proteins (advanced oxidation protein products (AOPP) and protein carbonyl groups (PC)), and DNA (8-hydroxy-D-guanosine (8-OHdG)) were analyzed in morbidly obese patients before and after bariatric surgery as well as in the healthy controls. The concentrations of 8-isoP, AOPP, PC, and 8-OHdG were significantly higher in both NS and S of patients with morbid obesity than in the control patients and compared to the results obtained 6 months after bariatric surgery. The levels of oxidative damage markers were also higher in S versus NS of morbidly obese patients. In summary, morbid obesity is associated with oxidative damage to salivary proteins, lipids, and DNA, while bariatric treatment generally lowers the levels of salivary oxidative damage.

## 1. Introduction

Overweight and obesity are chronic diseases characterized by excessive accumulation of adipose tissue. According to the WHO, at least 50% of adults and 20% of children are overweight (BMI > 25 kg/m^2^), and more than 400 million are obese (BMI > 30 kg/m^2^). Moreover, the number of people with morbid obesity (BMI > 40 kg/m^2^) has increased by 4-5 times compared to the 1990s. Complications of morbid obesity are extremely dangerous to human health and include metabolic syndrome, cardiovascular disease, insulin resistance (IR), and type 2 diabetes [1]. Treatment of morbid obesity requires multidirectional actions, but the most effective method is surgical treatment, including also bariatric surgery [2]. There are a number of surgical methods; however, the presented study only included laparoscopic gastric sleeve resection. This technique is well tolerated by patients, which allows for their faster recovery. There is a decrease in the number of postoperative complications, satisfactory reduction of body weight, and associated diseases [3].

Not only the aforementioned excess of the adipose tissue, but also, above all, its dysfunction is central to obesity-related complications. It was shown that adipose tissue in obese patients develops an inflammatory milieu, which drives oxidative stress (OS) due to a large increase in reactive oxygen species (ROS) formation by immune cells as part of the immune response [4, 5]. Oxidative stress is a situation in which chronically elevated ROS levels lead to disturbances in cellular metabolism and degradation of cellular components such as lipids, proteins, and DNA [4–6]. The cell membrane is the first element to be exposed to contact with free radicals; therefore, the earliest symptom of developing OS is lipid peroxidation. Most often the lipids with one or more double bonds are subject to oxidative modifications leading to the formation of peroxides (4-hydroxynonenal (4-HNE) and 8-isoprostanes (8-isoP)). Oxidative modifications of proteins and free amino acid residues are also observed at high ROS concentrations. Oxidation of proteins results in breaking of protein chains, formation of cross-links within single or multiple polypeptide chains, and modification of amino acid residues. Oxidative damage to proteins can be assessed by measuring concentrations of, for example, protein carbonyls (PC) or advanced oxidation protein products (AOPP). Oxidative DNA modifications are observed in a form of, for instance, increased concentrations of 8-hydroxy-D-guanosine (8-OHdG) [7, 8].

It is believed that OS causes damage to the salivary gland components and promotes chronic systemic and local inflammation. It leads, inter alia, to the initiation and progression of pathological changes within the oral cavity. Indeed, it was shown that more than half of people with morbid obesity are diagnosed with diseases of oral cavity, the pathogenesis of which may be associated with excessive ROS [9–11]. Moreover, there is evidence indicating that salivary gland dysfunction is already manifested at the obesity stage [10]. Taking into account the importance of saliva in maintaining oral homeostasis, it becomes clear that, starting with the obesity stage, IR and diabetes can adversely affect oral health and the quality of life. The pathogenesis of the salivary glands dysfunction in the course of morbid obesity is still unknown.

The influence of oxidative stress in the pathogenesis of salivary gland dysfunction in the course of insulin resistance or type 2 diabetes has been confirmed [7, 12, 13]. To the best of our knowledge, there are hardly any sources describing salivary OS in the course of morbid obesity.

The purpose of this experiment is to assess the occurrence and intensity of oxidative stress in unstimulated and stimulated saliva of patients with morbid obesity before and 6 months after bariatric surgery by evaluating the concentrations of oxidative damage markers of lipids, proteins, and DNA. The relationship between the oxidative stress markers and the secretory function of salivary glands in the course of morbid obesity and its treatment has not been studied so far. Our goal is to better understand the relationship between oxidative stress and salivary gland dysfunction in the course of morbid obesity.

## 2. Materials and Methods

The research was approved by the Bioethics Committee of the Medical University of Bialystok, Poland (permission number: R-I-002/175/2012 of 31 May 2012). Every patient was informed about the purpose of the study and consented in writing to participate in the project.

### 2.1. Patients

The study involved 47 patients with morbid obesity (14 men and 33 women, aged 34 to 55). Patients were treated in the 1st Clinical Department of General and Endocrine Surgery of the Medical University of Bialystok. From 2012 to 2014, the patients underwent bariatric surgeries (laparoscopic gastric sleeve resection) performed by the same qualified surgeon (H.R.H.). Immediately before and 6 months after bariatric surgery, each patient had a blood test and a dental examination, and their unstimulated and stimulated mixed saliva was collected. The control group (C) consisted of 47 healthy, age- and gender-matched adults under the supervision of the UMB Department of Restorative Dentistry. Dental examinations as well as the collection of unstimulated and stimulated salivary samples from the control group were performed on a one-off basis.

Criteria for inclusion in the study group were as follows:

- BMI > 40 kg/m^2^
- Waist circumference ≥ 94 cm in men and ≥80 cm in women
- Total cholesterol concentration ≥ 200 mg/dL
- LDL cholesterol concentration ≥ 160 mg/dL
- HDL cholesterol concentration < 40 mg/dL

Criteria for inclusion in the control group were as follows:

- BMI 18–25 kg/m^2^
- Total cholesterol concentration < 200 mg/dL
- LDL cholesterol concentration < 160 mg/dL
- HDL cholesterol concentration > 40 mg/dL

Criteria for inclusion in the study and control group were as follows:

- Age 34–55
- HOMA-IR (Homeostatic Model Assessment of Insulin Resistance) < 1
- Blood uric acid concentration 180–420 *μ*mol/L
- AspAT and ALAT activity < 510 nmol/L/s
- TSH concentration in serum 2–5 mU/L
- Lack of inflammatory condition in the mouth, including gingivitis (no bleeding while probing gingival pockets; pale pink gingivae) and periodontitis (PPD: periodontal pocket depth measured from the gingival margin to the pocket bottom < 4 mm [14]; CAL: clinical attachment loss < 3 mm)

Criteria for exclusion from the study and control group were as follows:

- Smoking
- Alcohol consumption (except occasional use, once every 2-3 months)
- Taking medicines (including antibiotics and glucocorticosteroids) and dietary supplements affecting saliva secretion and its antioxidant status in the last 3 months
- Comorbidities of inflammatory etiology and involvement of oxidative stress (including diabetes, hypertension, diseases of kidneys, liver, thyroid gland, gastrointestinal tract, and immune system)
- Pregnancy

### 2.2. Dental Examination

The dental examinations took place at the UMB Department of Restorative Dentistry. The examinations were performed by the same qualified dentist (A. Z.), in artificial light, by means of an explorer, a mirror, and a periodontal probe. The DMFT index (total number of decayed, missing, and filled teeth) was calculated in accordance with the criteria of the WHO [15] as well as the SBI (Sulcus Bleeding Index) [16], PPD, and CAL. The intrarater reliability for DMFT was *r* = 0.97, for SBI *r* = 0.96, for PPD *r* = 0.92, and for CAL *r* = 0.96.

### 2.3. Saliva Collection

The study material was mixed stimulated and unstimulated saliva. It was collected via the spitting method at least 2-3 hours after tooth brushing or food and fluid intake (except water), always between 8 a.m. and 10 a.m. Saliva collection was performed in one separate room for all the patients, without exposing them to the effects of additional aromatic, taste, and visual stimuli. The patients had their saliva collected in a comfortable seated position, with the head slightly inclined forward. After rinsing the mouth three times with distilled water, the patients spat the saliva gathered at the bottom of the oral cavity for 15 minutes in total. Saliva was collected into sterile centrifuge tubes placed in a container with ice. The saliva collected for the first minute was discarded [17]. Saliva secretion was stimulated with a 2% citric acid solution placed at the back of the tongue (100 *μ*L every 30 seconds). Stimulated saliva was collected for a total of 5 minutes [18]. The volume of unstimulated and stimulated saliva was measured with a pipette set to 100 *μ*L. The saliva minute flow was calculated by dividing the volume of the secreted saliva by the time needed for its collection. To prevent oxidation of the sample during processing and storage, the solution of butylated hydroxytoluene (BHT) (10 *μ*L 0.5 M BHT in acetonitrile per 1 mL of saliva) was added to saliva samples [19]. Immediately upon collection, the saliva samples were centrifuged at 12000 ×g (4°C). The supernatant, frozen to −80°C immediately after centrifugation, was maintained for biochemical determination or further study.

### 2.4. Biochemical Analysis

In samples of unstimulated and stimulated saliva, the concentration of lipid peroxidation products (4-HNE protein adducts and 8-isoprostanes (8-isoP)), protein oxidation products (advanced oxidation protein products (AOPP) and protein carbonyl groups (PC)), and DNA oxidation products (8-hydroxy-D-guanosine (8-OHdG)) was marked. All assays were performed in duplicate samples and standardized to 100 mg total protein.

The concentrations of 4-HNE protein adduct, 8-isoP, and 8-OHdG were determined by the ELISA method (enzyme-linked immunosorbent assay) using ready-made reagent kits (Cell Biolabs, Inc., San Diego, CA, USA; Cayman Chemicals, Ann Arbor, MI, USA; USCN Life Science, Wuhan, China, resp.) in accordance with the manufacturer's instructions. The coloured end-product absorption was measured at 450 nm wavelength using the Mindray Microplate Reader, Shenzhen, China.

AOPP concentration was determined by colorimetric method according to Kalousová et al. [20], measuring the total oxidation capacity of iodide ion. Immediately before the assay, saliva samples were diluted 1 : 5 (v : v) with PBS (pH 7.4). The absorbance was measured at 340 nm wavelength using the Infinite M200 Pro microplate reader, Life Science, Tecan.

PC concentration was determined by colorimetric method according to the Reznick and Packer method [21]. In the presence of 2,4-dinitrophenylhydrazine (2,4-DNPH), PC forms a stable complex connections having a maximum absorption at 355–390 nm wavelength. The absorption of the resulting complexes was measured at 360 nm wavelength using the Infinite M200 Pro microplate reader, Life Science, Tecan. The milimolar absorption coefficient for 2,4-DNPH (22 mM^-1 ^cm^−1^) was used to evaluate the PC content.

The total protein content was determined colorimetrically by the bicinchoninic method (BCA), using a ready-made reagent kit (Thermo Scientific Pierce BCA Protein Assay Kit, Rockford, IL, USA) and a bovine serum albumin standard (BSA).

### 2.5. Statistical Methods

Statistical analysis was performed using the Statistica 10.0 (Cracow, Poland). Due to the fact that the obtained results were characterized by normal distribution, the following parametric tests were used: ANOVA post hoc NIR test for comparing multiple groups and the *t*-test for comparing two groups. The Cohen Kappa (online calculator) was used to establish an intrarater agreement between the two measurements of the assessed dental indicators performed by one researcher at an interval of two days. Pearson correlation coefficients were used to determine the association between the two variables. The results are presented as mean ± SD. The assumed statistical significance was *p* < 0.05.

## 3. Results

### 3.1. General Characteristics of the Patients

Bariatric surgery returned the BMI, total cholesterol, LDL, HDL, and triglycerides to the values observed in the control group (Table 1). The values of the stomatological parameters did not differ between the control group and obese patients before and after bariatric surgery (Table 1). Bariatric surgery did not affect dental findings (Table 1).

Mean unstimulated salivary flow in morbid obese individuals was significantly lower compared to the normal weight control and 6 months after bariatric surgery (*p* = 0.001 and *p* = 0.01, resp.). Mean value of stimulated salivary secretion in morbid obese at the baseline and 6 months after surgery was significantly lower compared to control patients (*p* = 0.002 and *p* = 0.003, resp.) (Table 1). However, the mean protein concentration in the unstimulated and stimulated saliva of patients with morbid obesity prior to surgery was significantly lower in comparison with control patients (*p* = 0.03 and *p* = 0.01, resp.) and 6 months after bariatric surgery (*p* = 0.03 and *p* = 0.04, resp.) (Table 1).

### 3.2. Lipid Oxidation Products

**Figure 1:**
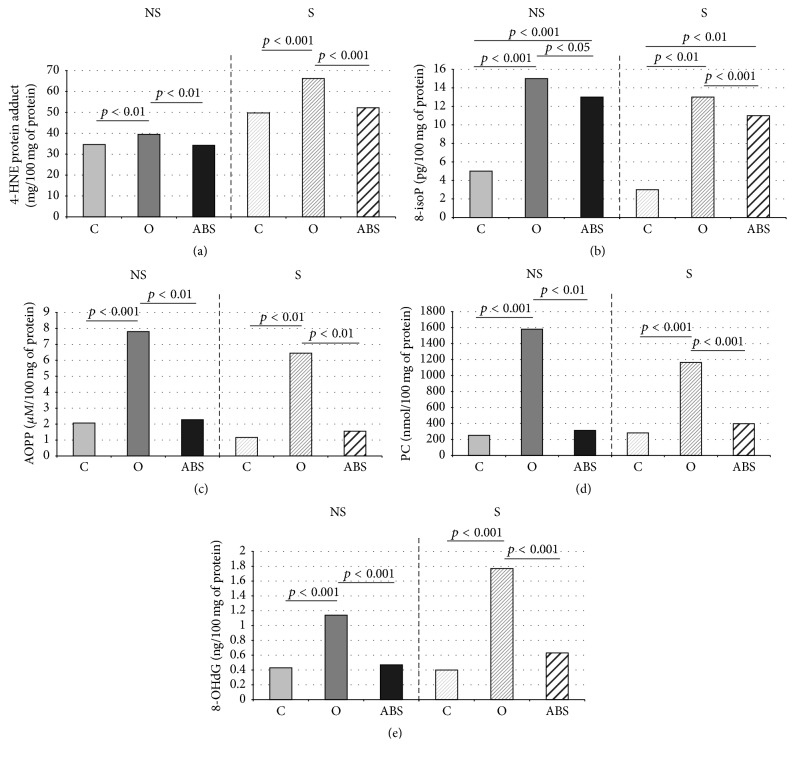
*Oxidative damage to lipids (a, b), proteins (c, d), and DNA (e) in unstimulated and stimulated saliva of patients with morbid obesity at the baseline and 6 months after bariatric surgery as well as the healthy controls*. C, control; O, morbid obese patients; ABS, patients after the bariatric surgery; NS, unstimulated whole saliva; S, stimulated whole saliva; 4-HNE protein adducts, 4-hydroxynonenal protein adducts; 8-isoP, 8-isoprostanes; AOPP, advanced oxidation protein products; PC, protein carbonyl groups, 8-OHdG, 8-hydroxyguanosine.

The mean 4-HNE-protein adduct concentration was significantly higher in unstimulated and stimulated saliva of patients with morbid obesity before the surgery compared to healthy controls (*p* = 0.003 and *p* = 0.001, resp.) and patients with morbid obesity 6 months after bariatric surgery (*p* = 0.0001 and *p* = 0.00001, resp.) (Figure 1(a)).

The mean 4-HNE-protein adduct concentration was significantly higher in stimulated compared to the unstimulated saliva in the control (*p* = 0.00001) as well as in morbid obese at the baseline (*p* = 0.000001) and 6 months after surgery (*p* = 0.000001) (Table 2).

The 8-isoP concentration in patients with morbid obesity prior to the surgery was significantly higher in both unstimulated and stimulated saliva compared to the controls (*p* = 0.0000001 and *p* = 0.001, resp.) and results obtained 6 months after bariatric surgery (*p* = 0.03 and *p* = 0.000001, resp.). 8-isoP concentrations in unstimulated and stimulated saliva in patients with obesity after bariatric surgery were significantly higher than in the control group (*p* = 0.0000001, *p* = 0.001, resp.) (Figure 1(b)).

The 8-isoP concentration was significantly higher in unstimulated compared to the stimulated saliva in the control (*p* = 0.000001) as well as in morbid obese at the baseline (*p* = 0.001) and 6 months after surgery (*p* = 0.0001) (Table 2).

### 3.3. Protein Oxidation Products

In the group of patients with morbid obesity prior to surgical treatment, the mean AOPP concentrations in both unstimulated and stimulated saliva were considerably higher than in the control patients (*p* = 0.00005 and *p* = 0.0009, resp.) and compared to the results obtained 6 months after bariatric surgery (*p* = 0.00009 and *p* = 0.002, resp.) (Figure 1(c)).

The mean AOPP concentration was significantly higher in the unstimulated saliva compared to the stimulated saliva of the control (*p* = 0.0006) and morbid obese patients 6 months after surgery (*p* = 0.0002) (Table 2).

In both unstimulated and stimulated saliva of morbidly obese patients, the mean PC concentration was significantly higher compared to the control group (*p* = 0.0000001 and *p* = 0.00000001, resp.) and results obtained 6 months after bariatric surgery (*p* = 0.001 and *p* = 0.0000001, resp.) (Figure 1(d)).

The mean PC concentration was significantly higher in the unstimulated saliva compared to the stimulated saliva of the morbid obese patients at the baseline (*p* = 0.00001) and it was significantly higher in the stimulated saliva compared to the unstimulated saliva of the morbid obese patients 6 month after surgery (*p* = 0.0001) (Table 2).

### 3.4. DNA Oxidation Products

The mean value of 8-OHdG concentration in unstimulated and stimulated saliva of patients with morbid obesity at the beginning of the experiment was considerably higher than in the control patients (*p* = 0.0000001 and *p* = 0.00000001, resp.) and compared to the results obtained 6 months after bariatric surgery (*p* = 0.0000001 and *p* = 0.0000001, resp.) (Figure 1(e)).

The mean 8-OHdG concentration was significantly higher in stimulated saliva compared to unstimulated saliva of the morbid obese patients at a baseline (*p* = 0.0002) and six months after bariatric surgery (*p* = 0.0001) (Table 2).

### 3.5. Correlations

There was a negative correlation between 4-HNE protein adduct and unstimulated and stimulated salivary flow of morbid obese patients (*p* = 0.02, *r* = −0.67 and *p* = 0.03, *r* = −0,54, resp.). There was a negative correlation between 8-isoP and stimulated salivary flow of morbid obese patients six months after bariatric surgery (*p* = 0.01, *r* = −0,69).

There was no correlation between DMFT, SBI, PPD, CAL, blood parameters and 4-HNE protein adduct, 8-isoP, AOPP, PC. and 8-OHdG concentrations in the unstimulated and stimulated saliva of morbid obese patients before and after bariatric surgery.

## 4. Discussion

This is the first study displaying that morbid obesity increased while its treatment with bariatric surgery generally decreases oxidative damage to lipids, proteins, and DNA both in unstimulated and in stimulated human saliva.

Saliva is a secretion from salivary glands that forms the environment of the oral cavity and is responsible for preliminary digestion of food, cleansing both mucous membrane and teeth as well as maintaining proper pH in the oral cavity. Moreover, saliva participates in both specific and nonspecific immune defense as well as showing very effective antioxidative systems that protect oral cavity environment from harmful effect of reactive oxidative species and reactive nitrogen species (RNS). The coexistence of ROS overproduction and impairment of antioxidative systems is called oxidative stress. It is believed that OS leads to damage to the components of salivary gland cells and enhances chronic systemic and local inflammatory condition (through the increase in the production of proinflammatory cytokines). Therefore, oxidative stress results in the initiation and progression of numerous pathological changes within the oral cavity which most commonly include caries, gingivitis, periodontitis, candidiasis, and dysfunction of salivary glands. The latter can be observed in form of changes in both quality and quantity of secreted saliva [9, 11, 22].

It was shown that, in more than 50% of persons with morbid obesity, the presence of oxidative stress-related oral cavity diseases can be observed [23, 24]. Interestingly, no studies confirming OS presence in the oral cavity in these patients can be found. According to the research by Knaś et al. [10], impairment of antioxidative systems in the saliva of morbid obesity patients as well as a normalizing effect of bariatric surgery on these conditions can be observed. Although the authors evaluated also MDA concentration, the results of their research fail to allow evaluating the scope and predicting the effects of oxidative stress in the oral cavity. Not only did the applied method of evaluating MDA concentration show a minor diagnostic value, but it also should be emphasized that the test of single redox biomarker in isolation has limited value in the diagnosis, staging, and prognosis of the oxidative stress-related human diseases. Numerous approaches of the measurement of oxidatively changed cellular components have been described so far. In this experiment we used the most common assessment to evaluate oxidative damage: oxidized lipids (8-isoP and 4-HNE protein adduct), proteins (AOPP and PC), and DNA (8-OHdG).

To begin with, it should be emphasized that we found no relationship between the concentration of the examined parameters of OS and the local inflammatory process and general parameters. According to the results, the changes observed in the unstimulated and stimulated saliva may be caused by the dysfunction of the salivary glands, regardless of general and local inflammatory processes.

It is well accepted that human parotid gland produces saliva mainly after stimulation while submandibular gland provides unstimulated saliva [25, 26]. Therefore, it is believed that disorders of the content or secretion of stimulated saliva reflect abnormalities in the function of parotid salivary gland. Analogically, disorders related to secretion of unstimulated saliva are related to the dysfunction of submandibular gland.

Our study showed that both unstimulated and stimulated saliva of morbidly obese patients were characterized by an increased concentration of 4-HNE protein adduct, 8-isoP, AOPP, PC, and 8-OHdG compared to the obtained data pertaining to unstimulated and stimulated saliva of normal weight control. A greater percentage increase in the concentration of the majority of the analyzed oxidative products in the stimulated (4-HNE protein adduct ↑33%, 8-isoP ↑295%, AOPP ↑456%, and 8-OHdG ↑342%) versus unstimulated saliva (4-HNE protein adduct ↑14%, 8-isoP ↑217%, AOPP ↑276%, and 8-OHdG ↑165%) may prove increased oxidative damage to parotid gland compared to submandibular gland in morbidly obese patients. This increase in oxidative damage in stimulated saliva may be related to a considerable insufficiency of antioxidative systems of parotid versus submandibular glands in morbidly obese patients described by Knaś et al. [10]. On the other hand, more intense oxidative damage observed in stimulated saliva versus unstimulated saliva of morbidly obese patients may be related to the observed by other researchers enhanced storage of adipocytes in the parotid parenchyma which is almost absent in submandibular glands [27]. Adipocytes by monocyte chemoattractant protein-1 (MCP-1) activate the influx of monocytes and their conversion into macrophages. Macrophages release cytokines TNF-*α*, IL-6, and IL-1*β* and thus inflammation develops. This leads to the activation of NADPH oxidase in the phagocytic cells and the enhanced formation of ROS, which in a seriously defected antioxidant barrier leads to the oxidative damage and dysfunction of the parotid gland.

Six months following the procedure we observed a significant decrease in BMI and the concentrations of total cholesterol, fractions of HDL, LDL, and triglycerides compared to the values observed in the control group. Complete therapeutic success was not accompanied by prevention from salivary oxidative damage and failed to restore redox balance in stimulated and unstimulated saliva to the values observed in control group. Six months following bariatric surgery we observed a maintaining increased 8-isoP concentration in both stimulated and unstimulated saliva compared to the control group. However, selectively increased 8-isoP concentrations prove that six months after bariatric surgery salivary glands are subject to lower intensity of the oxidative stress in comparison to preoperative status. It has been shown that the earliest symptom of the oxidative stress is lipid peroxidation since the lipids of cellular membrane are the first ones to be exposed to the harmful effects of free radicals. Only in case of an increase in the concentration of ROS, the concentration of lipid peroxidation products increases and proteins, and later DNA, undergo oxidation [28].

It should be stressed that our study showed a high degree positive effect of the loss of body mass in decreasing oxidative stress in unstimulated and stimulated saliva. The bariatric surgery related body loss protective action was noted in significantly lower concentrations of 4-HNE protein adduct, 8-isoP, AOPP, PC, and 8-OHdG in stimulated and nonstimulated saliva 6 months after the procedure compared to preoperative status. Furthermore, the concentrations of 4-HNE protein adduct, AOPP, PC, and 8-OHdG of both stimulated and unstimulated saliva showed the same values as the ones observed in the control group.

We observed a negative correlation between 4-HNE protein adduct and unstimulated and stimulated salivary flow of morbid obese patients. It is an interesting correlation considering the fact that 4-HNE protein adduct activates the expression of proinflammatory cytokines (TGF-*β*1 and IL-1) and metalloproteinases through ROS-mediated stimulation Akt-kappaB signaling pathway. TGF-*β*1 inhibits DNA synthesis and expression of Na^+^/K^+^ ATPase, both being essential for epithelial proliferation [29]. As a result, the loss of acinar cells and the exchange of parenchyma function with fibrous tissue occur. Inflammatory condition and reconstruction of extracellular matrix resulting from the effect of increased activity of metalloproteinases are a known factor that leads to a decreased response of residual acinar cells to Ach (acetylcholine), NA (noradrenaline), or receptor reconstruction. All these processes may lead to the reduced secretion of both stimulated and nonstimulated saliva and impaired mechanism involved in the synthesis/secretion of protein, the phenomena we observed in the saliva of morbidly obesity patients. Similarly, a negative correlation between 8-isoP concentration and stimulated secretion in patients 6 months after surgical procedure may be responsible for the fact that the secretion of stimulated saliva was significantly higher compared to preoperative values yet was also significantly lower compared to control group. Isoprostanes are a potential factor that impairs the integrity and liquidity of the mucous membrane as well as the function of membranous receptors [30].

There are certain limitations to our study. There are a number of different markers of ROS-related modification, yet our analysis included only those used most commonly. Using other markers of OS may partially or completely alter our observations and conclusions. Changes in oxidation marker concentrations may also result from increased postoperative intake of fruit and vegetables, as recommended by the surgeon, that are rich in polyphenols. The latter are known to stick to oral mucosa and increase salivary total antioxidative capability and thus prevent biomolecule oxidative modifications [13]. Undoubtedly, an advantage of this work is a relatively high number of patients carefully selected in terms of carbohydrate-lipid metabolism and accompanying diseases and the fact that this is the first study evaluating the scope of oxidative damage in the saliva of patients before and after bariatric surgery.

## 5. Conclusions

1. Oxidative modification of cellular components was greater in the stimulated saliva versus unstimulated saliva of morbidly obese patients.
2. Six months after bariatric surgery a decreased oxidative modification of biomolecules in unstimulated and stimulated saliva could be observed, yet bariatric surgery related weight loss was not effective in restoring redox balance in the oral cavity.
3. The presence of oxidative stress within salivary glands in morbidly obese patients may be indicative of the fact that antioxidant supplementation could decrease/remove hypofunction of salivary glands in this group of patients.

## Figures and Tables

**Table 1:** Demographic data, general health, stomatological findings, salivary flow, and protein concentration of the control and morbid obese patients at the baseline and 6 months after bariatric surgery (mean ± standard deviation).

	C (*n* = 47)	O (*n* = 47)	ABS (*n* = 47)
Male/female	14/33	14/33	14/33
Age	42.80 ± 13.10	44.52 ± 10.51	45.12 ± 11.11
BMI (kg/m^2^)	20.61 ± 2.31^*∗*^	47.10 ± 0.81^*∗∗*^	22.30 ± 3.4
TC (mg/dL)	147.51 ± 12.8^*∗*^	210.70 ± 10.05^*∗∗*^	160.3 ± 11.10
LDL (mg/dL)	105.70 ± 25.30^*∗*^	180.40 ± 15.81^*∗∗*^	119.30 ± 24.11
HDL (mg/dL)	43.23 ± 2.15^*∗*^	29.31 ± 1.12^*∗∗*^	38.23 ± 2.10
DMFT	20.50 ± 7	18.50 ± 9	18.50 ± 9
SBI	0.84 ± 0.10	0.9 ± 0.10	0.9 ± 0.10
CAL (mm)	2.7 ± 0.3	2.8 ± 0.5	2.8 ± 0.5
PPD (mm)	2.5 ± 1.1	2.7 ± 0.5	2.7 ± 0.5
NS (mL/min)	0.41 ± 0.10^*∗*^	0.28 ± 0.04^*∗∗*^	0.39 ± 0.17
S (mL/min)	1.21 ± 0.10^*∗*^	0.74 ± 0.20	0.76 ± 0.21^*∗∗∗*^
TP (NS) (mg/mL)	0.84 ± 0.01^*∗*^	0.64 ± 0.02^*∗∗*^	0.80 ± 0.05
TP (S) (mg/mL)	1.04 ± 0.25^*∗*^	0.75 ± 0.04^*∗∗*^	0.97 ± 0.22

C, control; O, morbid obese patients; ABS, patients after the bariatric surgery; BMI, body mass index; TC, total cholesterol; LDL, low density lipoprotein; HDL, high density lipoprotein; DMFT, decayed, missing, filled teeth; SBI, Sulcus Bleeding Index; CAL, clinical attachment loss; PPD, periodontal pocket depth; NS, unstimulated saliva secretion; S, stimulated saliva secretion; TP, total protein. ^*∗*^*p* < 0.05 C:O, ^*∗∗*^*p* < 0.05 O:ABS, ^*∗∗∗*^*p* < 0.05 ABS:C.

**Table 2:** Comparison of oxidative damage products in unstimulated and stimulated saliva of patients with morbid obesity before and after bariatric surgery as well as healthy controls.

	C		O		ABS	
	NS	S	NS	S	NS	S
4-HNE (*μ*g/100 mg of protein)	34.62 ± 8.80	49.72 ± 8.34	39.49 ± 6.92	66.17 ± 15.38	34.16 ± 7.94	52.22 ± 9.85
	**p** < 0.001		**p** < 0.001		**p** < 0.001	
8-isoP (pg/100 mg of protein)	4.65 ± 1.71	3.30 ± 0.90	14.76 ± 5.41	13.05 ± 3.14	12.80 ± 4.74	10.63 ± 2.12
	**p** < 0.001		**p** < 0.01		**p** < 0.001	
AOPP (*μ*mol/100 mg of protein)	2.07 ± 1.59	1.16 ± 0.69	7.80 ± 11.37	6.45 ± 13.07	2.28 ± 1.06	1.56 ± 0.75
	**p** < 0.001		*p* > 0.05		**p** < 0.001	
PC (nmol/100 mg of protein)	253.31 ± 103.25	282.76 ± 185.58	1579.51 ± 403.26	1164.21 ± 454.74	314.07 ± 104.20	398.32 ± 100.54
	*p* > 0.05		**p** < 0.001		**p** < 0.001	
8-OHdG (ng/100 mg of protein)	0.43 ± 0.19	0.40 ± 0.16	1.14 ± 0.83	1.77 ± 1.34	0.47 ± 0.22	0.63 ± 0.18
	*p* > 0.05		**p** < 0.001		**p** < 0.001	

C, control; O, morbid obese patients; ABS, patients after the bariatric surgery; NS, unstimulated whole saliva; S, stimulated whole saliva; 4-HNE protein adducts; 4-hydroxynonenal protein adducts; 8-isoP, 8-isoprostanes; AOPP, advanced oxidation protein products; PC, protein carbonyl groups; 8-OHdG, 8-hydroxy-D-guanosine.
